# Functional and Metabolic Correlates of Age- and Sex-Normalized Handgrip Strength in Children with Metabolic Dysfunction-Associated Steatotic Liver Disease

**DOI:** 10.3390/jcm15197519

**Published:** 2026-09-27

**Authors:** Mohit Kehar, Carolina Jimenez-Rivera, Leah Feret, Shelley E. Keating, Patricia E. Longmuir, Jonathan G. Stine

**Affiliations:** 1 Division of Gastroenterology and Hepatology, Department of Pediatrics, Children’s Hospital of Eastern Ontario, University of Ottawa, 401 Smyth Road, Ottawa, ON K1H 8L1, Canada; 2 School of Human Movement and Nutrition Sciences, The University of Queensland, Brisbane, QLD 4072, Australia; s.keating@uq.edu.au; 3 Children’s Hospital of Eastern Ontario Research Institute, Ottawa, ON K1H 8L1, Canada; 4 School of Human Kinetics, Faculty of Health Sciences, University of Ottawa, Ottawa, ON K1N 6N5, Canada; 5 Division of Gastroenterology and Hepatology, Department of Medicine, Penn State Health Milton S. Hershey Medical Center, Hershey, PA 17033, USA

**Keywords:** skeletal muscle, physical activity, functional status, pediatrics

## Abstract

**Background**: To characterize associations of age- and sex-normalized handgrip strength with physical activity, functional status, fatigue, metabolic parameters, and liver disease characteristics in children with metabolic dysfunction-associated steatotic liver disease (MASLD). **Methods**: This cross-sectional analysis included a prospectively recruited pediatric MASLD cohort. Maximum handgrip strength was measured using a Takei GRIP-A dynamometer following a protocol aligned with the Canadian Health Measures Survey and normalized using Canadian age- and sex-specific reference values. Low grip strength was defined as <25th percentile. Physical activity, functional status, and fatigue were assessed using the Physical Activity Vital Sign, Lansky Play-Performance Scale, and child- and parent-proxy PedsQL Multidimensional Fatigue Scale, respectively. **Results**: Fifty children were included (median age, 14.0 years; 70% male; median BMI z-score, 3.1). Low handgrip strength was present in 17/50 participants (34%). Normalized grip strength correlated inversely with controlled attenuation parameter (CAP; rho = −0.34, *p* = 0.024) and positively with HDL cholesterol (rho = 0.49, *p* < 0.001), weekly exercise (rho = 0.42, *p* = 0.003), and Lansky score (rho = 0.35, *p* = 0.012). Children with low grip strength had lower weekly exercise, Lansky scores, and HDL cholesterol. Median CAP was 318 dB/m in children with low grip strength and 296 dB/m in those without low grip strength (*p* = 0.085). **Conclusions**: Lower age- and sex-normalized handgrip strength was associated with lower physical activity, poorer functional status, lower HDL cholesterol, and higher CAP in children with MASLD. These findings support further study of handgrip strength as a simple functional measure in pediatric MASLD.

## 1. Introduction

Metabolic dysfunction-associated steatotic liver disease (MASLD) has become the most common chronic liver disease in children, closely paralleling the pediatric obesity epidemic [1,2,3,4]. Pediatric-onset disease is not uniformly benign. In a recent cohort of 1096 children followed for a mean of 8.5 years, 3.4% died, nearly half of deaths were liver-related, and the cumulative incidence of cirrhosis was 4.7%; dyslipidemia, hypertension, obstructive sleep apnea, and type 2 diabetes also developed at high rates [5]. Severe presentations, including hepatopulmonary syndrome, have also been reported [6]. These findings reinforce the need to characterize the full burden of pediatric MASLD.

Lifestyle management, including dietary intervention and regular physical activity (PA), is central to pediatric MASLD care [2,3,7,8]. Clinical phenotyping, however, remains weighted toward anthropometry, liver biochemistry, and noninvasive estimates of steatosis and fibrosis. These measures do not establish whether a child can initiate and sustain PA, has reduced muscle strength, or experiences fatigue that may interfere with treatment. Children with MASLD report impaired health-related quality of life and fatigue [9]. Our group has previously shown that, despite broad recognition that PA was important, fatigue and discomfort or pain was reported as barriers by 84% and 56% of children, respectively [10]. Functional limitation may therefore represent both an under-recognized component of MASLD burden and a barrier to its treatment.

Skeletal muscle provides a biologically plausible link between function and hepatic disease. It is a major site of insulin-mediated glucose disposal and participates in fatty-acid oxidation, myokine signaling, and systemic metabolic homeostasis. In MASLD, skeletal-muscle insulin resistance, myosteatosis, reduced muscle quantity or strength, and physical inactivity may reinforce hepatic lipid accumulation and systemic inflammation, while liver-derived signals may adversely affect muscle quality and function [11]. In chronic liver disease, frailty, sarcopenia, and malnutrition are overlapping but distinct constructs; recent pediatric literature emphasizes age- and sex-appropriate assessment, the risk of muscle deficits being obscured by obesity, and the need for feasible functional measures [12,13,14].

The pediatric muscle–liver literature remains limited and fragmented. Prior studies have related hepatic steatosis to relative muscle mass, body-composition measures, or handgrip strength, but functional status, habitual activity, fatigue, metabolic health, and liver phenotyping have not been examined together in a real-world, well-characterized pediatric MASLD cohort [15,16,17,18,19,20,21,22,23]. We therefore evaluated age- and sex-normalized maximum handgrip strength together with PA, Lansky PPS, multidimensional fatigue, metabolic markers, and vibration-controlled transient elastography (VCTE) measures in children followed in a dedicated pediatric MASLD clinic. We hypothesized that lower normalized grip would be associated with greater hepatic steatosis, less favorable metabolic health, lower PA, poorer functional status, and greater fatigue.

## 2. Methods

### 2.1. Study Design and Population

This was a cross-sectional analysis of children enrolled in a prospective single-center pediatric MASLD study at the Children’s Hospital of Eastern Ontario. Between September 2025 and June 2026, consecutive eligible patients attending the pediatric MASLD clinic were approached for participation. Nine eligible patients declined participation; 50 consented and were enrolled. No enrolled participant was subsequently excluded, and all 50 were included in the analysis. MASLD was defined by hepatic steatosis documented on clinically obtained imaging, including ultrasonography and/or VCTE CAP, in the presence of cardiometabolic risk and after exclusion of competing liver diseases and significant alcohol exposure, consistent with pediatric guidance [3]. The study was approved by the Children’s Hospital of Eastern Ontario Research Ethics Board (REB 20250340), conducted in accordance with the Declaration of Helsinki, and written informed consent and age-appropriate assent were obtained. The study is reported in accordance with the Strengthening the Reporting of Observational Studies in Epidemiology (STROBE) statement [24] (see Supplementary Materials).

### 2.2. Clinical and Anthropometric Data

Baseline demographic, clinical, anthropometric, laboratory, VCTE, biopsy, medication, and comorbidity data were collected from study records. Variables included age, sex, weight, height, body mass index (BMI), BMI z-score, waist circumference, blood pressure, screen time, race/ethnicity, reason for MASLD evaluation, comorbidities, and medication exposure. Race/ethnicity and reasons for evaluation were not mutually exclusive.

### 2.3. Assessment of Physical Activity, Functional Status, and Fatigue

PA was assessed using the Physical Activity Vital Sign/Exercise Vital Sign framework during a structured interview administered by trained clinic research staff, with responses provided by the child and clarified by a parent or caregiver when needed [25]. Minutes of moderate-to-vigorous activity per active day and active days per week were recorded. Weekly exercise was calculated for each participant as minutes per active day multiplied by active days per week. At least 420 min/week was used to approximate the World Health Organization recommendation of an average of 60 min/day of moderate-to-vigorous activity for school-aged children and adolescents [26]. Functional status was assessed with the Lansky PPS, a 0–100 observational pediatric performance scale originally developed for children with cancer [27]. A score of 100 denotes full normal activity, 80 an active child who tires more quickly, 60 a child who is up and around but has minimal active play and mainly quieter activities, and 50 a child who lies around much of the day but can dress and participate in quiet activities. Higher scores indicate better function. AASLD frailty guidance identifies the Lansky PPS as a promising global functional-status measure for children with liver disease, and the scale has been associated with outcomes in pediatric liver and lung transplant candidates [12,28,29].

Fatigue was assessed using separate PedsQL Multidimensional Fatigue Scale child self-report and parent-proxy forms. Children completed the self-report and caregivers completed the proxy form independently; the two reports were analyzed separately, with child–parent agreement and paired differences assessed as secondary analyses. The 18-item instrument evaluates general, sleep/rest, and cognitive fatigue and has been validated in pediatric obesity and used in pediatric liver disease [30,31]. Responses were transformed to a 0–100 scale (Never = 100, Almost never = 75, Sometimes = 50, Often = 25, Almost always = 0), and domain and total scores were calculated as the mean of completed items. Higher scores indicate less fatigue and better functioning. The instrument was used under permission from ePROVIDE/Mapi Research Trust.

### 2.4. Handgrip Strength Assessment

Grip strength was measured in kilograms by trained clinic research staff using a Takei GRIP-A analog grip-strength dynamometer (T.K.K. 5001; Takei Scientific Instruments Co., Ltd., Niigata, Japan) with standardized verbal instructions and encouragement. The testing procedure was aligned with the Canadian Health Measures Survey (CHMS): participants stood with feet slightly apart and held the dynamometer in line with the forearm, away from the body at thigh level. Two maximal efforts were obtained from each hand in alternating order, with approximately 30 s between successive attempts on the same hand. Participants were instructed to squeeze as hard as possible while exhaling. The highest value attained by each hand was retained, and maximum grip strength was defined as the highest value obtained from either hand [32]. Because handgrip strength changes rapidly with growth and differs by sex, maximum grip strength was interpreted against CHMS age- and sex-specific reference values [32,33]. Age at assessment was matched to the nearest whole-year reference category. For the secondary categorical analysis, low grip was defined as maximum grip strength below the age- and sex-specific 25th percentile. This evidence-informed threshold was selected because P25 or lowest-quartile definitions have been used in pediatric studies relating grip strength to adiposity, body composition, and cardiometabolic risk, using sex-specific, age- and sex-specific, or sex- and weight-specific approaches [34,35,36,37,38]. A systematic review of pediatric sarcopenic obesity documents the use of percentile-based definitions while also demonstrating substantial heterogeneity across studies [39]; reviews of health-related youth muscular-fitness cut-points and pediatric sarcopenia similarly emphasize the absence of universal diagnostic cut-points [40,41]. Adult MAFLD/NAFLD studies have similarly shown that the lowest sex-specific quartile of relative grip strength is associated with steatosis or advanced fibrosis [42,43]. No P25 threshold has been validated specifically in pediatric MASLD. Accordingly, the P25 category was treated as secondary and exploratory and describes low normalized grip rather than a diagnosis of sarcopenia; inference was prioritized from continuous normalized grip. Normalized grip was calculated as observed maximum grip strength divided by the corresponding age- and sex-specific median (50th percentile), multiplied by 100; 100% therefore represents the reference median. Percentile bands below the 10th and 5th percentiles were also described to characterize the distribution and severity of low values.

### 2.5. Liver and Metabolic Assessment

Laboratory measures included alanine aminotransferase (ALT), aspartate aminotransferase (AST), gamma-glutamyl transferase (GGT), albumin, international normalized ratio (INR), ferritin, creatinine, platelets, fasting insulin, fasting glucose, lipid profile, and hemoglobin A1c. Homeostatic Model Assessment for Insulin Resistance (HOMA-IR) was calculated as fasting glucose (mmol/L) multiplied by fasting insulin (microIU/mL) divided by 22.5, with insulin converted from pmol/L using 1 microIU/mL = 6.945 pmol/L. VCTE (FibroScan, Echosens, Paris, France) was performed by trained clinic personnel according to manufacturer recommendations using an appropriate probe. CAP was used as an imaging-based measure of hepatic steatosis burden and liver stiffness as a noninvasive marker of fibrosis risk [44]. Clinically available biopsy findings were summarized descriptively.

### 2.6. Statistical Analysis

Continuous variables were summarized as median (IQR), and categorical variables as n/N (%). Study size was determined by the number of consecutive eligible participants who consented and completed the standardized assessments during the recruitment period; no formal a priori sample-size calculation was performed. The primary analyses used age- and sex-normalized grip as a continuous variable. Spearman rank correlations assessed associations between normalized grip and liver, metabolic, activity, functional, and fatigue measures. Secondary exploratory analyses included P25 group comparisons, logistic regression, and the additional fatigue and PPS correlations reported in Table S2. Because the study was hypothesis-generating, no formal multiplicity adjustment was applied; all reported *p*-values are unadjusted. Mann–Whitney U tests compared participants below and at or above P25. Child–parent fatigue agreement was assessed using Spearman correlation, and paired child–parent differences were evaluated using Wilcoxon signed-rank testing. Logistic regression examined low grip using clinically interpretable scaling: CAP per 50 dB/m, HDL cholesterol per 0.1 mmol/L, weekly exercise per 100 min, PPS per 10 points, BMI z-score per unit, and waist circumference per 10 cm. Given 17 low-grip events, regression was restricted to univariable analyses and two post hoc two-variable models to limit overfitting [45]. Weekly exercise was selected as the sole covariate because PA could plausibly relate to both muscle performance and the CAP and HDL associations of interest. The regression models were descriptive and were not intended to establish prediction or independent associations. All statistical analyses were performed using Stata, version 19.5 (StataCorp LLC., College Station, TX, USA).

## 3. Results

### 3.1. Cohort Characteristics

All 50 enrolled children and adolescents with MASLD were included in the analysis, with no post-enrollment exclusions. Median age was 14.0 years (IQR 11.7–15.8), 35/50 (70%) were male, median BMI was 33.9 kg/m^2^ (IQR 29.5–36.9), and median BMI z-score was 3.1 (IQR 2.7–3.5) (Table 1). Median waist circumference was 107.8 cm (IQR 100.0–117.5; *n* = 45). The most frequently reported race/ethnicity categories were White (28/50, 56%), Middle Eastern (11/50, 22%), Indigenous (5/50, 10%), South Asian (4/50, 8%), and Southeast Asian (3/50, 6%). MASLD evaluation was most prompted by an incidental finding (30/50, 60%) or screening in the setting of weight gain (20/50, 40%).

### 3.2. Physical Activity, Functional Status, and Fatigue

Median moderate-to-vigorous PA was 60 min per active day (IQR 30–60) over 5 active days/week (IQR 4–5), while the participant-level median weekly total was 210 min/week (IQR 150–360) (Table 1). Only 11/50 (22%) achieved at least 420 min/week. Median Lansky PPS was 75 (IQR 60–80); 38/50 (76%) had PPS ≤ 80 and 22/50 (44%) had PPS ≤ 60. Median child-reported total fatigue score was 52.8 (IQR 44.4–62.2), and median parent-proxy total score was 51.4 (IQR 44.4–64.9) (Table 1). Child–parent agreement was strong for total fatigue (rho = 0.80, *p* < 0.001) and sleep/rest fatigue (rho = 0.85, *p* < 0.001). Children reported greater sleep/rest fatigue than caregivers perceived (median 54.2 vs. 58.3; *p* = 0.002). Internal consistency was good for children (Cronbach alpha = 0.87) and parent-proxy (alpha = 0.88) scales.

### 3.3. Handgrip Strength Distribution

The right hand was dominant in 44/50 participants (88%). Median maximum grip strength was 25.25 kg (IQR 17.5–33.0) (Table 1). Using the exploratory P25 threshold, low grip was present in 17/50 (34%), including 14/35 males and 3/15 females (Fisher *p* = 0.209). Eleven participants (22%) were below the 10th percentile, including nine (18%) below the 5th percentile (Figure 1; Table S1).

### 3.4. Biochemical, Elastography, and Biopsy Findings

Median ALT was 48 U/L (IQR 29.5–91.5; *n* = 48), AST 37 U/L (IQR 24–54; *n* = 46), GGT 32 U/L (IQR 22–50; *n* = 45), HDL cholesterol 1.12 mmol/L (IQR 0.99–1.21; *n* = 46), and HOMA-IR 7.0 (IQR 4.0–9.5; *n* = 45). VCTE was available in 44/50 participants (88%). Median CAP was 315.5 dB/m (IQR 287.0–334.5), and median liver stiffness was 6.0 kPa (IQR 5.25–7.0). Liver stiffness ≥ 8 kPa was present in 8/44 (18.2%), and none had liver stiffness ≥ 15 kPa. CAP ≥ 300 dB/m was present in 27/44 (61.4%). Liver biopsy was clinically available in four participants, with fibrosis stage 2–3 and no biopsy-reported cirrhosis. The small biopsy subset precluded meaningful comparison with VCTE findings.

### 3.5. Associations with Normalized Grip

In continuous analyses, normalized grip correlated inversely with CAP (rho = −0.34, *p* = 0.024) and positively with HDL cholesterol (rho = 0.49, *p* < 0.001), weekly exercise (rho = 0.42, *p* = 0.003), and Lansky PPS (rho = 0.35, *p* = 0.012) (Table 2; Figure 2). It was not associated with ALT, BMI z-score, waist circumference, liver stiffness, child total fatigue, or parent total fatigue. Raw maximum grip correlated strongly with age (rho = 0.68), height (rho = 0.71), weight (rho = 0.57), and creatinine (rho = 0.72; all *p* < 0.001), supporting use of age- and sex-referenced rather than raw grip values.

### 3.6. Low-Grip Comparisons and Exploratory Regression

In secondary exploratory comparisons, participants below the P25 threshold had lower weekly exercise (150 vs. 300 min/week, *p* = 0.017), lower Lansky PPS (60 vs. 80, *p* = 0.043), and lower HDL cholesterol (0.99 vs. 1.15 mmol/L, *p* = 0.012) than participants with preserved grip. Median CAP was higher in participants below P25 than in those at or above P25 (318 vs. 296 dB/m), although the difference did not reach statistical significance (*p* = 0.085). ALT, GGT, BMI z-score, waist circumference, liver stiffness, and fatigue scores did not differ between groups (Table 3). In post hoc exploratory univariable logistic regression, the HDL estimate was below one (OR 0.59 per 0.1 mmol/L, 95% CI 0.38–0.91; *p* = 0.016), whereas the CAP confidence interval included the null (OR 2.45 per 50 dB/m, 95% CI 0.95–6.30; *p* = 0.063). Weekly exercise and Lansky PPS estimates were also imprecise. In two-variable models including weekly exercise, the CAP confidence interval continued to include the null (adjusted OR 2.42, 95% CI 0.89–6.62; *p* = 0.085); the HDL estimate remained below one (adjusted OR 0.59, 95% CI 0.38–0.92; *p* = 0.021) (Table 4).

### 3.7. Fatigue and Functional Associations

In secondary exploratory analyses, higher fatigue scores indicate less fatigue. Child total fatigue correlated inversely with waist circumference (rho = −0.44, *p* = 0.003), screen time (rho = −0.29, *p* = 0.047), and HOMA-IR (rho = −0.34, *p* = 0.022), and positively with PPS (rho = 0.35, *p* = 0.014). Parent total fatigue correlated inversely with waist circumference (rho = −0.40, *p* = 0.006), screen time (rho = −0.35, *p* = 0.015), and liver stiffness (rho = −0.35, *p* = 0.021), and positively with PPS (rho = 0.40, *p* = 0.004). Exploratory PPS correlations are reported in Table S2. *p*-values were not adjusted for multiple comparisons.

## 4. Discussion

In this prospectively recruited cohort of 50 children with MASLD, reduced handgrip strength was common: 34% had a maximum grip strength below the age- and sex-specific 25th percentile, and more than one-fifth were below the 10th percentile. In continuous analyses, lower normalized grip strength was consistently associated with lower PA, poorer functional status, lower HDL cholesterol, and higher CAP. Comparisons using the P25 threshold demonstrated a similar pattern, with lower weekly exercise, Lansky PPS, and HDL cholesterol among children with low grip strength. Together, these findings establish a consistent within-cohort pattern linking lower muscle strength with functional and metabolic measures in pediatric MASLD. To our knowledge, this is the first pediatric MASLD study to combine standardized handgrip dynamometry with VCTE, metabolic phenotyping, PA, functional status, and both child- and parent-reported fatigue in the same children. These findings support further evaluation of handgrip dynamometry as a practical complementary measure of physical function in pediatric MASLD.

Evolving data suggest that skeletal muscle is an active component of MASLD biology rather than simply a marker of illness. It is a major site of insulin-mediated glucose disposal and fatty-acid oxidation and participates in myokine, inflammatory, and substrate signaling to the liver [11]. Reduced muscle insulin sensitivity and quality may increase glucose and lipid delivery to the liver, while hepatic and adipose signals may further impair muscle metabolism. Shared ectopic fat deposition, insulin resistance, inflammation, and physical inactivity provide a plausible framework linking greater hepatic steatosis with poorer muscle function. The relationship between muscle strength and liver disease has received increasing attention in adults [46,47,48], but pediatric evidence remains limited [49]. Song et al. studied 337 Korean children and adolescents and found that grip-to-weight and grip-to-BMI ratios were inversely associated with MASLD; grip-to-weight achieved an AUC of approximately 0.71 [15]. In a clinical cohort of 100 adolescents with MASLD, Lee et al. reported associations between grip measures and selected laboratory values in males when grip was indexed to body weight [16]. A population-based analysis of 1690 Korean adolescents further suggested that higher age-, sex-, and weight-standardized grip attenuated the association of obesity and metabolic syndrome with MASLD [17]. These studies support the relevance of muscle strength but used relative strength indices influenced by body size and did not integrate functional status, fatigue, and VCTE. Our use of age- and sex-specific Canadian reference values provides a complementary approach that interprets muscle performance against expected values during growth and development.

Our results also complement pediatric body-composition studies. Yodoshi et al. linked lower psoas muscle area on imaging and lower impedance-derived relative muscle mass with more severe hepatic steatosis in pediatric MASLD [18,19]. Pacifico et al. similarly reported an association between low relative muscle mass and MASLD in youth with overweight or obesity [20]. In adolescents with MASLD, Kwon et al. found that lower muscle-to-fat ratio tracked with greater ultrasound-graded disease severity and less favorable cardiometabolic indices [21]. More recent work has associated lower DXA-derived appendicular lean mass index with hepatic steatosis in adolescents with overweight or obesity and identified sex-specific relationships between muscle-to-fat balance and pediatric MASLD [22,23]. Our results extend these observations by demonstrating that a simple bedside measure of muscle function parallels these imaging-based findings. Unlike body-composition techniques, handgrip dynamometry requires minimal equipment, can be incorporated into routine clinic visits within minutes, and reflects functional muscle performance rather than muscle quantity alone.

Normalized handgrip strength was not associated with BMI z-score or waist circumference in this cohort, which had a median BMI z-score of 3.1. The concentration of participants within the severe-obesity range limited the ability to evaluate relationships across different levels of adiposity. The observed differences in strength, PA, and functional status may reflect the combined contributions of severe obesity, deconditioning, and MASLD. Studies spanning a broader adiposity range and including children with obesity without MASLD will help clarify these contributions.

Continuous normalized grip correlated inversely with CAP (rho = −0.34, *p* = 0.024), whereas ALT was not associated with grip strength. This difference is biologically plausible because CAP estimates hepatic fat, while ALT reflects hepatocellular injury and may fluctuate independently of steatosis. The CAP finding is also consistent with pediatric body-composition studies linking lower relative muscle mass with greater steatosis [18,22,23]. Median CAP was higher in the P25 low-grip group (318 vs. 296 dB/m), although the categorical comparison did not reach statistical significance (*p* = 0.085). The CAP regression estimates were directionally consistent, but their confidence intervals crossed the null. The continuous and categorical results are not contradictory: dichotomizing grip strength reduces information and statistical power, particularly with only 17 participants below P25. Accordingly, the evidence for an association with hepatic steatosis in this cohort comes from the continuous CAP analysis rather than from a validated P25 risk threshold. Longitudinal studies incorporating MRI-PDFF will help define the magnitude and temporal significance of this relationship. The predominantly ambulatory, non-cirrhotic profile of the cohort may also explain the absence of an association with liver stiffness.

The PA and functional-status findings reinforce the clinical relevance of the grip-strength results. Participants below P25 reported less weekly exercise and had lower median Lansky PPS scores than participants with preserved grip strength. AASLD guidance identifies the Lansky PPS as a promising global measure of pediatric function, and the scale has been associated with outcomes in pediatric liver and lung transplant candidates [28,29]. The concordance among objectively measured grip strength, PA, and Lansky PPS is consistent with reduced functional reserve in ambulatory children with MASLD. Because the P25 subgroup contained 17 participants and the comparisons were not adjusted for multiple testing, the threshold requires validation in larger cohorts.

Fatigue adds a related but distinct patient-centered dimension to the functional assessment. Consistent with our previous work [10], both children and caregivers reported fatigue, with child- and parent-reported total scores near the midpoint of the scale. Children also reported greater sleep/rest fatigue than their caregivers perceived. The differing relationships of fatigue and grip strength with other functional measures support their interpretation as complementary rather than interchangeable constructs: grip strength measures physical performance, whereas fatigue captures the child’s perceived burden. Because the supplementary fatigue and PPS correlations were not adjusted for multiple comparisons, these associations require confirmation in larger cohorts.

HDL cholesterol was the most consistent metabolic correlate of grip strength across both continuous and P25 analyses. This finding aligns with pediatric fitness literature associating higher grip strength with lower odds of borderline or low HDL cholesterol [50] and with Canadian population data linking greater handgrip strength to a more favorable cardiometabolic profile [33]. When weekly exercise was included in the post hoc two-variable model, the HDL estimate remained essentially unchanged, demonstrating consistency across analyses. Given the limited number of low-grip participants, however, this model was designed to assess consistency rather than establish an independent association. The relationship is biologically coherent because HDL may reflect habitual PA, insulin sensitivity, and skeletal-muscle oxidative capacity rather than directly determining grip strength. It may also be relevant to MASLD outcomes, as lower HDL independently predicted mortality in a recent long-term pediatric MASLD cohort [5]. Prospective studies should determine whether interventions that improve muscle strength also improve HDL cholesterol and hepatic steatosis.

A major strength of this study is its comprehensive participant-level phenotyping across domains rarely evaluated together in pediatric MASLD. All 50 participants completed standardized assessments of handgrip strength, PA, functional status, and fatigue; VCTE was available in 44/50 (88%), and laboratory phenotyping was nearly complete. This enabled integrated evaluation of muscle strength alongside hepatic, metabolic, functional, and patient-reported measures. The study was limited by its single-center, cross-sectional design, cohort size, and the inclusion of 17 participants below P25. Without an obesity-only comparator, the relative contributions of MASLD, severe obesity, and deconditioning cannot be separated. The concentration of participants within the severe-obesity range also limited evaluation of associations across different levels of adiposity. The P25 threshold has empirical precedent in pediatric strength research but has not been validated specifically in pediatric MASLD; in this study, it represents a low-strength category rather than a diagnosis of sarcopenia. Because the analyses were hypothesis-generating, *p*-values were not adjusted for multiple comparisons and were interpreted according to effect direction, magnitude, consistency, and precision. The event-limited post hoc logistic models were descriptive and were not designed to establish prediction or independent associations. Additional limitations include limited histological confirmation, self-reported PA, and the absence of direct measures of muscle quantity or composition.

These findings provide a framework for multicenter longitudinal validation. Future studies should include children with obesity without MASLD and determine whether continuous normalized grip strength tracks with longitudinal changes in MRI-PDFF or CAP, liver stiffness, cardiometabolic risk, fatigue, and responses to exercise or weight-management interventions. Combining handgrip assessment with DXA, bioelectrical impedance analysis, muscle ultrasound, or MRI-based measures could help distinguish muscle quantity, muscle quality, and deconditioning. MASLD-specific diagnostic or prognostic thresholds can then be derived and externally validated before categorical grip measures are applied in clinical classification.

In conclusion, lower age- and sex-normalized handgrip strength was associated with lower PA, poorer functional status, lower HDL cholesterol, and higher CAP in children with MASLD and severe obesity. The P25 analysis identified a subgroup with lower activity, Lansky PPS, and HDL cholesterol, while the association with CAP was supported by the continuous analysis rather than the categorical comparison. Together, these findings position handgrip strength as a promising, simple functional measure for further study in pediatric MASLD. Multicenter longitudinal studies incorporating obesity-only comparators and direct measures of hepatic fat and muscle composition are now warranted.

## Figures and Tables

**Figure 1:**
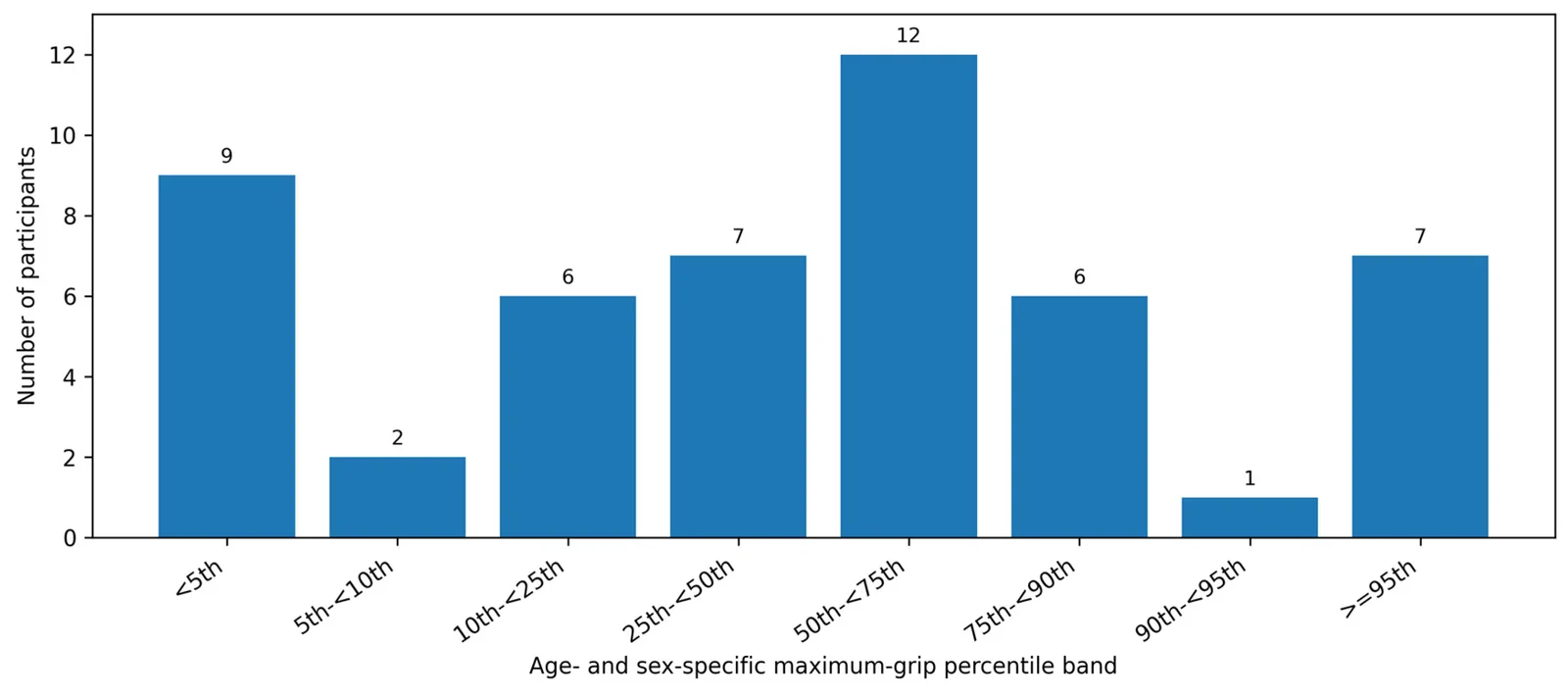
Age- and sex-specific maximum-grip percentile distribution.

**Figure 2:**
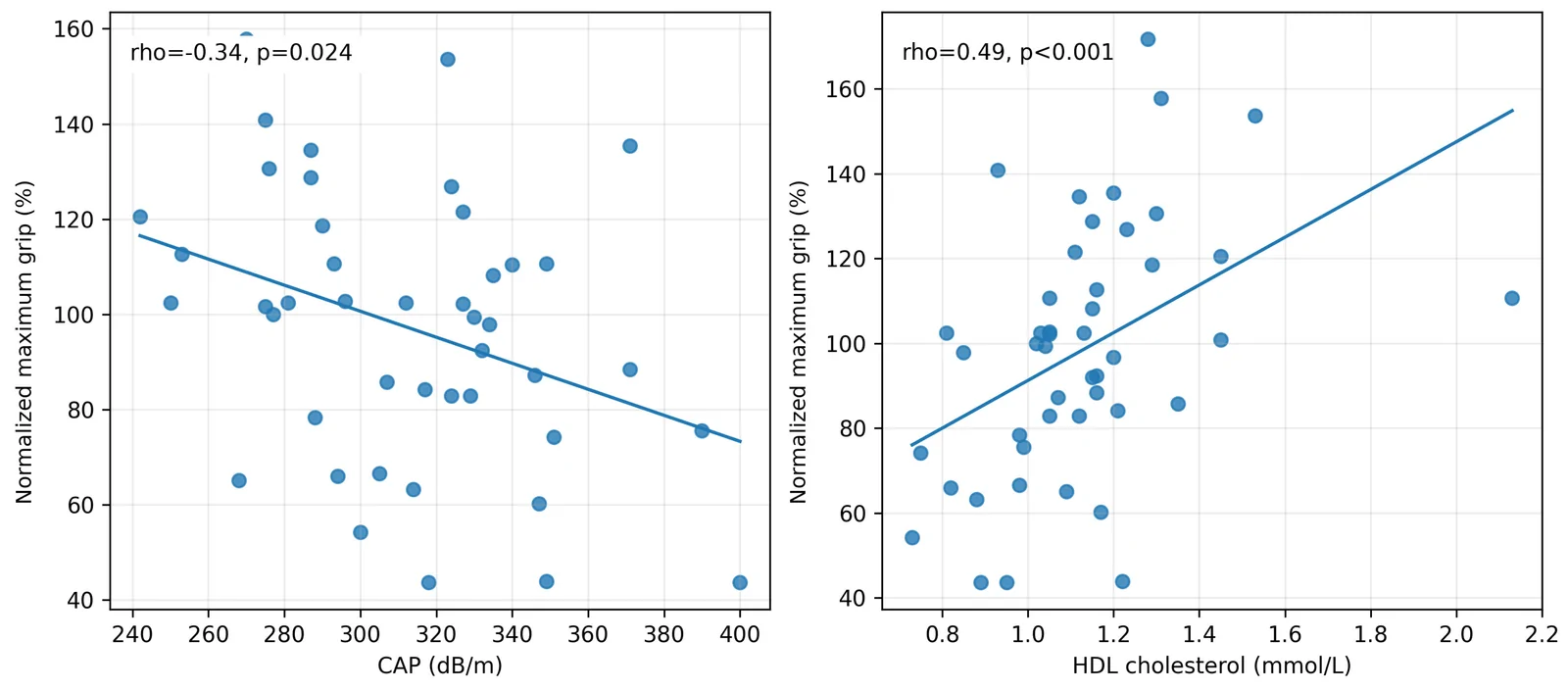
Age- and sex-normalized maximum handgrip strength versus CAP and HDL cholesterol.

**Table 1:** Baseline cohort characteristics (*N* = 50).

Variable	Result, Median (IQR) or *n*/*N* (%)
Age, years	14.0 (11.7–15.8), *n* = 50
Male sex	35/50 (70.0%)
Weight, kg	91.2 (74.2–105.0), *n* = 50
BMI, kg/m^2^	33.9 (29.5–36.9), *n* = 50
BMI z-score	3.1 (2.7–3.5), *n* = 50
Waist circumference, cm	107.8 (100.0–117.5), *n* = 45
Screen time, h/day	4.0 (2.2–6.0), *n* = 47
Weekly exercise, min/week	210 (150–360), *n* = 50
Achieved ≥ 420 min/week	11/50 (22.0%)
Lansky PPS	75 (60–80), *n* = 50
PPS ≤ 80	38/50 (76.0%)
PPS ≤ 60	22/50 (44.0%)
Maximum grip strength, kg	25.25 (17.5–33.0), *n* = 50
Normalized grip, % of age/sex median	100.4 (82.8–112.6), *n* = 50
Low grip (<25th percentile)	17/50 (34.0%)
Child total fatigue score	52.8 (44.4–62.2), *n* = 50
Parent total fatigue score	51.4 (44.4–64.9), *n* = 50
ALT, U/L	48 (29.5–91.5), *n* = 48
HDL cholesterol, mmol/L	1.12 (0.99–1.21), *n* = 46
CAP, dB/m	315.5 (287.0–334.5), *n* = 44
Liver stiffness, kPa	6.0 (5.25–7.0), *n* = 44

Note. ALT, alanine aminotransferase; BMI, body mass index; CAP, controlled attenuation parameter; HDL, high-density lipoprotein; IQR, interquartile range; PPS, Play-Performance Scale.

**Table 2:** Continuous age- and sex-normalized maximum handgrip strength correlations.

Variable	Rho	*p*-Value
CAP, dB/m	−0.34	0.024
HDL cholesterol, mmol/L	0.49	<0.001
Weekly exercise, min/week	0.42	0.003
Lansky PPS	0.35	0.012
ALT, U/L	−0.09	0.539
BMI z-score	−0.01	0.959
Waist circumference, cm	0.00	0.978
Liver stiffness, kPa	0.07	0.632
Child total fatigue score	0.23	0.114
Parent total fatigue score	0.19	0.178

Note. BMI, body mass index; CAP, controlled attenuation parameter; HDL, high-density lipoprotein; PPS, Play-Performance Scale. *p*-values were not adjusted for multiple comparisons.

**Table 3:** Exploratory low-grip versus preserved-grip comparisons, median (IQR).

Variable	Low Grip (<25th Percentile)	Preserved Grip (≥25th Percentile)	*p*-Value
CAP, dB/m	318 (305–347), *n* = 17	296 (276–332), *n* = 27	0.085
HDL cholesterol, mmol/L	0.99 (0.89–1.12), *n* = 17	1.15 (1.05–1.28), *n* = 29	*0.012*
Weekly exercise, min/week	150 (100–210), *n* = 17	300 (150–420), *n* = 33	*0.017*
Lansky PPS	60 (50–80), *n* = 17	80 (60–90), *n* = 33	*0.043*
ALT, U/L	49 (34–90), *n* = 17	45 (26.5–95), *n* = 31	0.682
GGT, U/L	41 (28–51), *n* = 17	28.5 (21.5–48.2), *n* = 28	0.325
BMI z-score	3.06 (2.86–3.37), *n* = 17	3.07 (2.62–3.47), *n* = 33	0.927
Waist circumference, cm	104.0 (97.0–116.8), *n* = 15	108.2 (102.0–116.9), *n* = 30	0.605
Liver stiffness, kPa	5.4 (4.6–6.5), *n* = 17	6.1 (5.5–7.0), *n* = 27	0.148
Child total fatigue	52.8 (43.1–54.2), *n* = 17	56.9 (45.8–63.9), *n* = 33	0.277
Parent total fatigue	51.4 (41.7–61.1), *n* = 17	51.4 (45.8–66.7), *n* = 33	0.384

Note. ALT, alanine aminotransferase; BMI, body mass index; CAP, controlled attenuation parameter; GGT, gamma-glutamyl transferase; PPS, Play-Performance Scale. *p*-values were not adjusted for multiple comparisons. Italicized numerical *p*-values indicate unadjusted *p* < 0.05.

**Table 4:** Post hoc exploratory logistic regression for low maximum handgrip strength.

Predictors	OR	95% CI	*p* Value
**Univariable models**			
CAP, per 50 dB/m	2.45	0.95–6.30	0.063
HDL cholesterol, per 0.1 mmol/L	*0.59*	*0.38–0.91*	*0.016*
Weekly exercise, per 100 min/week	0.72	0.50–1.04	0.080
Lansky PPS, per 10 points	0.68	0.46–1.01	0.059
BMI z-score, per 1 unit	1.25	0.50–3.09	0.631
Waist circumference, per 10 cm	0.94	0.69–1.29	0.706
**Multivariable model 1: CAP + weekly exercise**			
CAP, per 50 dB/m (adjusted)	2.42	0.89–6.62	0.085
Weekly exercise, per 100 min/week (adjusted)	0.70	0.49–1.02	0.063
**Multivariable model 2: HDL + weekly exercise**			
HDL, per 0.1 mmol/L (adjusted)	*0.59*	*0.38–0.92*	*0.021*
Weekly exercise, per 100 min/week (adjusted)	0.80	0.57–1.11	0.175

Note. OR, odds ratio; CI, confidence interval; CAP, controlled attenuation parameter; HDL, high-density lipoprotein; PPS, Play-Performance Scale. Models were post hoc and event-limited. *p*-values were not adjusted for multiple comparisons. Bold row labels identify model groups. Italicized numerical results indicate associations with unadjusted *p* < 0.05.

## Data Availability

Deidentified data may be available from the corresponding author on reasonable request, subject to institutional and research ethics requirements.

## Supplementary Materials

The following supporting information can be downloaded at: https://www.mdpi.com/article/10.3390/jcm15197519/s1, Table S1: Age- and sex-specific maximum handgrip strength percentile distribution; Table S2: Exploratory correlations of fatigue and Lansky Play-Performance Scale scores with clinical, metabolic, and liver measures.
