# Supplementary material for: Functional and Metabolic Correlates of Age- and Sex-Normalized Handgrip Strength in Children with Metabolic Dysfunction-Associated Steatotic Liver Disease
Source: J Clin Med. 2026 Sep 27;15(19):7519. doi: 10.3390/jcm15197519 (PMC13666077; doi:10.3390/jcm15197519)
Supplement: Supplementary file 1 [file jcm-15-07519-s001.zip › jcm-4559128-supplementary.pdf]

Table S1. Age- and sex-specific maximum handgrip strength percentile distribution.

Canadian age- and sex-specific maximum-grip percentile band	n/N (%)
<5th	9/50 (18.0%)
5th-<10th	2/50 (4.0%)
10th-<25th	6/50 (12.0%)
25th-<50th	7/50 (14.0%)
50th-<75th	12/50 (24.0%)
75th-<90th	6/50 (12.0%)
90th-<95th	1/50 (2.0%)
>=95th	7/50 (14.0%)

Table S2. Exploratory correlations of fatigue and Lansky Play-Performance Scale scores with clinical, metabolic, and liver measures. Higher PedsQL fatigue scores indicate less fatigue/better functioning; negative correlations therefore indicate worse fatigue with higher values of the correlate. PPS, Play-Performance Scale.

Outcome	Correlate	rho	p-value
Child total fatigue	Waist circumference	-0.44	0.003
Child total fatigue	Screen time	-0.29	0.047
Child total fatigue	HOMA-IR	-0.34	0.022
Child total fatigue	Lansky PPS	0.35	0.014
Parent total fatigue	Waist circumference	-0.40	0.006
Parent total fatigue	Screen time	-0.35	0.015
Parent total fatigue	Liver stiffness	-0.35	0.021
Parent total fatigue	Lansky PPS	0.40	0.004
Lansky PPS	Weekly exercise	0.35	0.014
Lansky PPS	Normalized grip	0.35	0.012
Lansky PPS	HDL cholesterol	0.31	0.039
Lansky PPS	HOMA-IR	-0.30	0.047

STROBE Statement Checklist

Functional and Metabolic Correlates of Age- and Sex-Normalized Handgrip Strength in Children with Metabolic Dysfunction-Associated Steatotic Liver Disease

Section	Item	STROBE recommendation	Location
Title and abstract	1	Indicate the study’s design with a commonly used term in the title or abstract.	p. 1 (Abstract identifies the cross-sectional design)
Title and abstract	2	Provide an informative and balanced summary of what was done and what was found.	pp. 1–2
Introduction	3	Explain the scientific background and rationale for the investigation being reported.	pp. 2–3
Introduction	4	State specific objectives, including any prespecified hypotheses.	p. 2
Methods	5	Present key elements of study design early in the paper.	pp. 2–3
Methods	6	Describe the setting, locations, and relevant dates, including periods of recruitment, exposure, follow-up, and data collection.	p. 3 (single-center setting; September 2025–June 2026 recruitment)
Methods	7	Give eligibility criteria and the sources and methods of selection of participants.	p. 3
Methods	8	Clearly define all outcomes, exposures, predictors, potential confounders, and effect modifiers. Give diagnostic criteria, if applicable.	pp. 3–5
Methods	9	For each variable of interest, give sources of data and details of methods of assessment; describe comparability if there is more than one group.	pp. 3–5
Methods	10	Describe any efforts to address potential sources of bias.	pp. 3–5 (standardized assessments); p. 12 (limitations)
Methods	11	Explain how the study size was arrived at.	p. 4 (consecutive recruitment determined N; no formal a priori calculation)
Methods	12	Explain how quantitative variables were handled in the analyses. If applicable, describe which groupings were chosen and why.	pp. 4–5
Methods	13a	Describe all statistical methods, including those used to control for confounding.	pp. 4–5
Methods	13b	Describe any methods used to examine subgroups and interactions.	p. 5 (secondary P25 comparisons and post hoc two-variable models)
Methods	13c	Explain how missing data were addressed.	pp. 5–9; Tables 1–4
Methods	13d	If applicable, describe analytical methods taking account of sampling strategy.	Not applicable;
Methods	13e	Describe any sensitivity analyses.	Not applicable; no sensitivity analyses were performed
Results	14a	Report numbers of individuals at each stage of the study.	pp. 3 and 5 (59 approached, 9 declined, 50 enrolled/analyzed; no post-enrollment exclusions)

Section	Item	STROBE recommendation	Location
Results	14b	Give reasons for non-participation at each stage.	p. 3 (9 eligible patients declined; no post-enrollment exclusions)
Results	14c	Consider use of a flow diagram.	Not used; participant flow is reported in the text on p. 3
Results	15a	Give characteristics of study participants and information on exposures and potential confounders.	pp. 5–6; Table 1
Results	15b	Indicate number of participants with missing data for each variable of interest.	pp. 5–9; Tables 1–4 (variable-specific denominators reported)
Results	16	Report numbers of outcome events or summary measures.	pp. 5–9; Tables 1–4; Figures 1–2
Results	17a	Give unadjusted estimates and, if applicable, confounder-adjusted estimates and their precision; make clear which confounders were adjusted for and why.	pp. 6–9; Tables 2–4
Results	17b	Report category boundaries when continuous variables were categorized.	pp. 4 and 6–8; Table S1 (p. 15)
Results	17c	If relevant, consider translating estimates of relative risk into absolute risk for a meaningful time period.	Not applicable
Results	18	Report other analyses done, e.g., subgroup, interaction, and sensitivity analyses.	pp. 8–9; Tables 3–4; Table S2 (p. 16)
Discussion	19	Summarize key results with reference to study objectives.	pp. 9–10
Discussion	20	Discuss limitations, taking into account sources of potential bias or imprecision; discuss direction and magnitude of potential bias.	p. 12
Discussion	21	Give a cautious overall interpretation considering objectives, limitations, multiplicity of analyses, similar studies, and other relevant evidence.	pp. 9–12
Discussion	22	Discuss the generalisability (external validity) of the study results.	p. 12
Other information	23	Give the source of funding and the role of the funders for the present study and, if applicable, the original study.	p. 13 (Funding statement)
